# SOCIAL DETERMINANTS OF HEALTH AND WILLINGNESS FOR END-OF-LIFE DISCUSSION AMONG KOREAN AMERICAN IMMIGRANTS

**DOI:** 10.1093/geroni/igac059.769

**Published:** 2022-12-20

**Authors:** Hyunjin Noh, Eunyoung Choi, Lewis Lee, Hee Lee

**Affiliations:** The University of Alabama School of Social Work , Tuscaloosa, Alabama, United States; School of Global Public Health, New York University, Los Angeles, California, United States; School of Social Work, The University of Alabama, Tuscalossa, Alabama, United States; University of Alabama, Birmingham, Alabama, United States

## Abstract

This study aimed to examine social determinants of health (SDH) associated with Korean American (KA) immigrants’ willingness for end-of-life discussions with family and doctors. A self-administered, cross-sectional survey was conducted with 259 KAs in Alabama. Demographic, health, acculturation, and SDH information were collected. Binary logistic regression analyses were conducted to examine associations between SDH and willingness for end-of-life discussion. Most participants were willing to discuss with family (94%) and doctors (82%). Those aware of hospice care were more likely to have willingness for discussion with family and doctors. Those who could not see a doctor because of cost and who had higher threats to interpersonal safety were less likely to have willingness for discussion with family. Those with more chronic conditions and higher social isolation were less likely to have willingness for discussion with doctors. Interventions aimed to promote KAs' end-of-life discussions should consider the SDH identified in this study.

